# Remaining Human in COVID-19: Dialogues on Psychogeography

**DOI:** 10.1007/s42087-021-00233-y

**Published:** 2021-06-11

**Authors:** Johanna L. Degen, Gemma Lucy Smart, Rosanne Quinnell, Kieran C. O’Doherty, Paul Rhodes

**Affiliations:** 1 Department of Psychology, European University Flensburg, Flensburg, Germany; 2 School for History and Philosophy of Sciences, University of Sydney, Sydney, Australia; 3 School of Life and Environmental Sciences, University of Sydney, Sydney, Australia; 4 Department of Psychology, University of Guelph, Guelph, ON Canada; 5 Clinical Psychology Unit, University of Sydney, Sydney, Australia

**Keywords:** COVID-19, Viralscape, Psychogeography, Crystallization, Dialogical Inquiry, Interdisciplinary Methods, Experimental Methods, Walking, Group Healing, Group Coping

## Abstract

Post-COVID-19 environments have challenged our embodied identities with these challenges coming from a variety of domains, that is, microbiological, semiotic, and digital. We are embedded in a new complex set of relations, with other species, with cultural signs, and with technology and venturing further into an era that pushes back on our anthropocentrism to create a post-human dystopia. This does not imply that we are less human or forfeit ethics in this state of flux, but can lead to considering new ways of being alive and humanists. The aim of this project was to explore walking through our associated psychogeographies as captured in photographs and text from individual walks, as the means by which to characterize responses to the distress of the pandemic and to assess resistance to non-being. The psychogeographies were the starting points for our dialogic enquiry between authors who each represent living theory, representing their own emergent knowledge, inseparable from personal commitments and history. Walking and the associated images and reflections, provided a way to regulate our affect, reconnecting with our bodies, leading to understand and adapt to new meanings of context and ways of coping and healing in this new becoming. The interdisciplinarity of philosophy, social psychology, botany, and clinical psychology is nonetheless rejected in favour of multi-vocality; each author representing their own emergent, living theory, inseparable from personal commitments, and history.

## Introduction

### Mapping Distress in the Viralscape

We are surrounded by small-scale life forms. They live on us, they live inside of us. Many are critical to our lives, some are harmless or even beneficial, and others are opportunistic pathogens. Certainly, since the time of Pasteur, but in particular now, it is the pathogenicity of microbes that attracts the attention of humans. We predominantly notice the negative impacts of their presence. At the very moment on a global scale, a pathogen, a specific virus, has our attention. Some posit that viruses are not alive (Forterre, 2010); however, alive or not, their reproduction relies on a host. How viruses are described and classified is informed by and in turn informs their pathogenic characteristics. COVID-19 infection disables the host’s immune system to mount a balanced response, which can lead to death.

This virus presents some peculiar and particular challenges, given the shifting of our relations including our physical bodies: to the microbiological, other animal species, culture, the digital world, and each other. Not only are we under existential threat from infection and the loss of loved ones, we also face ontological challenges— a change in the conduct of everyday life—that we need to try and understand and negotiate.

Psychological perspectives that focus primarily on the intrapsychic conditions and processes of the individual can hardly respond adequately to these new conditions, because they miss the theoretical foundation to recognize the individual as located in a nexus of biological and social relations, well beyond genetic and environmental determination, cognitive processes or supposedly rational decisions. Even cybernetic practices, such as systemic (family) therapy (Rhodes & Wallis, 2011), are similarly limited, given that they do not venture beyond interpersonal dynamics to our relations with other animate and inanimate objects.

*"Just like assemblages, affect is socio‐material and decentred: ‘affect is distributed between, and can happen outside, bodies which are not exclusively human, and might incorporate technologies, things, non‐human living matter, discourses.”*

*(Lorimer, *2008*, 552)*.

Our distress in the current conditions might instead be better understood through post-humanist inquiry, which considers the existential threats posed by a wider range of new and complex relations that are constantly in becoming, widening the horizon in both regard to theory and method. Research can then explore what multi-species and trans-corporeal (Alaimo, 2012) realities are emerging as a result of the present pandemic, in other words our deep entanglement with a broad range of phenomena in a constantly changing world.

In mapping distress, we use posthumanism as a guiding perspective alongside three contexts: the viralscape, the biosemiosphere, and the technosphere. All three contexts help us grasp the abrupt changes of the rupture in everyday life due to the impact of SARS-CoV-2. To help us think about mapping a context for this distress, we replace the bio-psycho-social model of distress with the microbiological-semiotic-technological.

### Context 1: The Viralscape

In the viralscape, we are now hyper-conscious of the unseen and the omnipresent and the others’ body. The viralscape has changed the unconscious sphere of cultural signs incorporated in a larger notion of world and meaning, where the meanings of specific objects and nature as a whole have been changed. Central to the present challenges is our embeddedness within what Arregui (2020) calls the “inter-corporeal scenery” we inhabit, whereby the border of the body has become more permeable than imagined, radically porous, under zoonotic transmission, the process where a pathogen is passed between humans and animals (Arregui, 2020; Lainé, 2018), blurring the perceived and constructed line of humans being different, or even superior, to other life and impacting habits, habitus, and the relation between bodies as virus carriers.

Of course, we have known this for some time. Research in the field of the human microbiome has emphasized that we have never been biologically distinct and exclusively “human” in the sense that we cohabit a space with countless colonies of microbes. By cell count, microbial cells outnumber human cells. On a genomic level, there are up to 1000 times more microbial genes associated with our bodies than there are genes in the human genome (Institute for Genome Sciences, 2020). In contrast to human genes, microbial genes can be transmitted horizontally as well as vertically; that is, microbial genes need not be disseminated by being passed onto “offspring,” they can be passed on to “friends.” In contrast to human cells, microbes can easily travel beyond the construed boundary of our skin. We share microbes with each other on a daily basis: every time we shake hands, kiss, and share a gym space. When we are born, our microbiome is seeded by our mother’s microbes, and our microbial identity is immediately influenced by whether we are exposed to vaginal microbes or skin microbes, depending on whether we were born via cesarean or not (Dominguez-Bello et al., 2010; LifHolgerson et al., 2011). In a very tangible way and in a biological sense, we are and always have been inter-connected and we are not as separate and autonomous as we sometimes may feel. Viruses are, in a very real way, part of the multiplicitous assemblages of which humans form a part, moving across space and time, with relational ties between us, and to objects and places beyond us (Müller & Schurr, 2016). Such assemblages are symbiotic and co-functioning (Deleuze & Parnet, 1987).

The SARS-CoV-2 virus operates on this microbial level, but in doing so also disrupts and changes our lives on a macro level. Under the spread of the illness, COVID-19 not only the human-animal relation has and will change but also the human–human relation. These inter-corporeal sceneries or “scapes” turn the microbial life of human and nonhuman others into a social concern (Arregui, 2020). Arregui argues that viruses are wild beings seizing bodies as their habitat, thus should be respected just as prey should be respected by their hunters. These microbial “social” dynamics also impact “human–human and human-nonhuman bodily engagements reshuffle viral relations themselves in unpredictable ways” (Arregui, 2020).

### Context 2: The Biosemiosphere

This virus has breached bodily borders, and the organismal hierarchy, where humans rule, has been overthrown. It is fascinating that Lotman (1984), cultural historian and originator of the term semiosphere, drew on the life sciences, specifically on Vernadsky’s concept of the biosphere (1945) to reflect the practices of “meaning making” within cultural environments. The term semiosphere refers to the collection of cultural signs we incorporate into ourselves, hidden to many, representing the hermeneutic environment we live in, and, articulating the fundamental questions of our attachment to art, literature, images, memes, etc. In the essay “On the Semiosphere” (1984), Lotman offers: “we justify our term [semiosphere] by analogy with the biosphere, as Vernadsky defined it, namely the totality and the organic whole of living matter and also the condition for the continuation of life” (1990, p. 125). The interactive sphere of sign process, meaning, and interpretation is deeply related to the natural world. Lotman’s legacy is extensive, and the role of biology in it is marginal and small. However, looking at it more carefully, we find that the biological part, a biologicity in the sense of biological holism, is nevertheless surprisingly important; it exists in considerable amounts (notably from the 1980s); and although the texts in which he expresses his views on more biological issues were mostly initiated by other people […], they may have been quite necessary for Lotman himself. In any case, he was open toward the biological direction of semiotics (Lotman, 1989, p. 127).

The virus, SARS-CoV-2, as a visual sign, takes on a cultural life that fits with this materialist view of semiotics as taken up by (Ponzio & Petrilli, 2001) and Merrel [sic], (2001). Since the onset of the virus, we have been cultivated to interpret the iconic red spiky image not simply as a pandemic with real medical and economic costs, but also as an apocalyptic one, an end-times hyperimage (Baudrillard, 1993). It takes its place alongside repeated images of ISIS beheadings on YouTube, CGI New York Disaster movies, and drowned children on the beach, in the semiotics of doom (Roberts & Cremin, 2017). The repetition of these signs becomes our cultural unconscious, a hidden affective atomos-fear (Tateo, 2019) that is triggered by the virus, converting appropriate anxiety to apocalyptic dread.

### Context 3: The Technosphere

The technosphere in this regard is not emerging in novelty, rather it is massively accelerated, changing from being an alternative and additional vehicle to a predominant social place “cutting” of the body. For instance, in therapy, where situatively being together changed to be in a communicative asynchronous technological channeled exchange of cognitive processes. It replaces physical cohesion and the idea of place and connectivity with a dislocated reachability and equality of the other. It does this while crossing borders into the most private aspects of lives, and without even noticing the geographically embedded some- “body.” Our being is under threat from the digital world. We have been herded onto Zoom by SARS-CoV-2 losing status as “somebodies,” no longer personified in flesh, in the absence of carnal intersubjectivity (Merleau-Ponty, 1960, 1964). Barlow described his first experience of virtual reality as “my everything has been amputated” (Tripathi, 2005, n.p.). While scientists claim that movement can be simulated through cognition, this may trick the brain and the simulation but will rarely trick the body (Leeb et al., 2006). In the new Zoo(m) environment, our body, our biggest sense organ, is excluded from our communications as we work, socialize, celebrate, and commiserate behind digital bars. The body, as Reggie Ray puts it, “is the unconscious, not only in the smaller but also in the largest sense. The body is ultimately our largest person” (The Buddhist Review, 2010, para. 1). Zoom-relations are exhausting because our mind is tricked into thinking we are together but we are not. As Petriglieri has tweeted “It is easier being in each other’s presence, or in each other's absence, than in the constant presence of each other’s absence” (Petriglieri, 2020).

This disembodiment within the digital web, however, is only one small feature in a much larger Technosphere (Haff, 2014), the post-industrial networked and dynamic system of machines that is an iconic trope for science fiction. Haff (2014), however, goes into great detail to describe the rules by which we are no longer just one-sided causal creators of, but also part of these systems, impacted by them (Schraube, 2009) and dependent on them for survival; technology here comes as materialized action, interconnecting subjectivity, sociability, and materiality (Schraube, 2009). This dependency is accelerated by the onset of COVID-19, exaggerating the development into an ontology to which we become subordinate, leaving the task of “the rehabilitation or reconstruction of psychological theory and conceptual categories to better account for technoscientific processes” (O’Doherty et al., 2019, p. 20). There is an urge for a disciplinary implementation of a “new-ish” focus: tech-psychology (O’Doherty et al., 2019).

### How to Remain Human

Posthumanism conjures up images of The Walking Dead, with herds of humans-without-agency, without ethics, stumbling through post-apocalyptic cities. Hurley (1996) writes that the “abhuman subject is a not-quite-human subject, characterized by its morphic variability, continually in danger of becoming not-itself, becoming other” (168) and becoming inhuman. The question remains: will we become the *abhuman subject—*the ontic-hauntic objects (Lauro & Embry, 2008) in the face of these new non-binary landscapes, or be able to retain and regain our humanity in the face of new positioning in a shattered and reforming assemblage? And how to?

Braidotti (2013) and Brinkmann (2017) argue, however, that posthumanism must not employ anti-humanism or the loss of ethics, but can be interpreted and lived as the return of the subject to new non-binary landscapes and providing hope in a dystopian apocalyptic scenery. Humans become (again) one species alongside others, not superior or in control, yet still capable and responsible to stabilize the unstable, which following Brinkmann (2017) is their specifically. A specificity directing to reveal patterns, regulating chaos, making "human lives, families, organizations, communities, and societies possible.” (Brinkmann, 2017, p. 124) in all forms.

1. We followed Brinkmann’s (2017) methodological approach of acknowledging humanist approaches within the post-humanist paradigm to refuse apocalyptic antihumanism. Post-humanism rightfully relegates humans as simply one amongst multiple species, without superiority. Retaining humanism within it, however, allows for the assertion of a de-centered responsibility and ethics (Brinkmann, 2017). Humanist posthumanism acknowledges our capacity to struggle towards the respectful coexistence of both human–human, human-animal, and human-nature relationship (Brinkmann, 2017).
2. We engaged in posthumanist inquiry with the aim of overcoming conventional hierarchies, like that between subject/object, internal/external (Brinkmann, 2017), a theoretical playground that allows for novelty, not mechanical science.
3. We engaged in posthumanist inquiry with the aim of capturing knowledge production beyond language. As Porpora explains “certainly, language use is part of what is traditionally associated with humanism, but perception, motor control, and even to an extent common sense move us out of representation and back to non-representative, bodily knowledge" (Porpora, 2017, p. 362).
4. We engaged in posthumanist inquiry with the aim of studying complex relations as they interact in the moment; a material way of thinking and being (Ulmer, 2017).
5. We reaffirmed humanity after the death of humanism.

### Remaining Human Together Apart: Connecting Through Walking and Dialogue

Under the distress and isolation of the pandemic, we gathered in the technosphere as colleagues and friends from three continents—a group of authors from varied disciplines: clinical and social psychology, philosophy, history and philosophy of science, human geography, and botany—to make sense, to understand the meaning of this new microbiological-semiotic-technological context from a polyphonic, intersubjective perspective. Our dialogic approach followed Bakhtin’s (1963) notion of polyphony, foregrounding the active role that speakers play in selecting among inner discourses and building continuous meaning in heteroglossic dialogue (Steinby & Tintti, 2013). This is not to say that we aim to predominantly represent, embody, our disciplines in this regard, but rather to conduct the inquiry as embodied persons whose professional commitments mirror long-standing histories and new becomings. We developed this paper as a joint journey of exploration in an uncertain stage of radical change, relying on our material-emotional, atheoretical knowledge, rather than on a priori theories and reflexive knowledge (Seikkula et al., 2006). We interacted and connected with each other, in writing, design and analysis, making theory not only a living thing, but one that is always co-evolving between people-physical location, history, education, notion of world, and state of mind collectively grounding a conjunctive truth (Mannheim, 1964).

## Method

Our inquiry had two phases; the first was a psychogeography/schizocartography of our respective local neighbourhoods, recorded both visually and in text. The second was a dialogical inquiry, in which we collectively interpreted the emerging meanings of the first phase. In our dialogical inquiry, the psychogeographies served as projective surfaces, as impulses for intersubjective introspections, reflections and growth of meaning, making the implicit explicit. We aimed through this dialogue to witness and contribute to each other's meaning-making and allow it to influence our own, engaging in iterative conversation without aiming for synthesis, finalization or morality (Bakhtin, 1981, 1984).

### Data Collection: Schizocartography

We are to resist the abhumanising forces (Slavkova, 2013) of isolation, separation, and standing still in the new viral-semiotic-technosphere. One can see many ways in which this has happened in lock-down, with a renaissance of gardening, bread-making, stitching, and bee-keeping, each of which attempts to reaffirm materiality and community. Instead we might start to find ways to resist this dread and disembodiment that threaten the ruination of the human subject, while acknowledging the physical reality of our humbled status through staying connected and moving our body, making inner voices explicit. To bring forth the intuitive, bodily knowledge, we rely on the method of “rhythmic walking” as a way to access sensing place, and to gain new corporeal insights about meaning and its aesthetics (Matos Wunderlich, 2008). Often unnoticed as being a routinized everyday practice it is at the same time a way to “immerse ourselves and dwell in the representational and lived world” (Matos Wunderlich, 2008, p. 3) and becomes both the mundane and surprising. Walking as a kind of “WalkingLab” (Matos Wunderlich, 2008, p. 2) aims to collectively examine vital, sensory, and ephemeral material as an intersubjective practice in collaboration with other scientists, artists, and online hubs for their creation (Matos Wunderlich, 2008). When and while moving the body intentionally through the world (Matos Wunderlich, 2008) one knows that movement not only facilitates but also contributes to cognition and brain activity (Leismann et al., 2016; Hamacher et al., 2015). While moving through the world, subjects feel through haptic sense their context. A context as a part of a global touch, manifesting as a bodily experience and emotion (Matos Wunderlich, 2008; Springgay & Truman, 2017), and sense of coherence with, for instance, stress reducing effects (Ikeda et al., 2021). In this study, we concern ourselves specifically with the method of walking and being in the outside world, recognizing that to be moved back into life, we must move. This is given by the inseparability of motion and emotion^1^ (Fuchs & Koch, 2014) and the potential enlivening value of integrating internal and external landscapes. This does, paradoxically, assimilating (back) into the human by the discipline of steps and breath and allows us to then interact in a more differentiated fashion across the transcorporeal. As psychogeographer Will Self puts it:

“I’ve taken to long-distance walking as a means of dissolving the mechanised matrix which compresses the space–time continuum, and decouples human from physical geography. So this isn’t walking for leisure – that would be merely frivolous, or even for exercise – which would be tedious.” (Self, 2003, n.p.)

We can use the term schizocartography (Richardson, 2015), following (Deleuze & Guatarri, 1987) in that the act of walking allows us to retain subjectivity while mapping our relations within the viral/cultural and technological realms. This is not solely an act of introspection, but rather the embodiment of our engagement with the conditions of the new assemblage under the pandemic.

We have operationalized our method using the mobile app, PolarSteps, to record images and responses to our respective walks. Our walks were undertaken in our local neighbourhoods in Germany, Canada, and Australia in the first week of May, 2020.

Our instructions were as follows:

1. Leave your front door
2. Turn on the App PolarSteps
3. Walk around your neighbourhood for about 1 h
4. Stop and take a photo with the App when you are moved by internal thoughts as they are triggered
5. Write a memo of your thoughts which will accompany the photo and be tagged by the app to the precise geographic place
6. Go home and close the front door

**Fig. 1:**
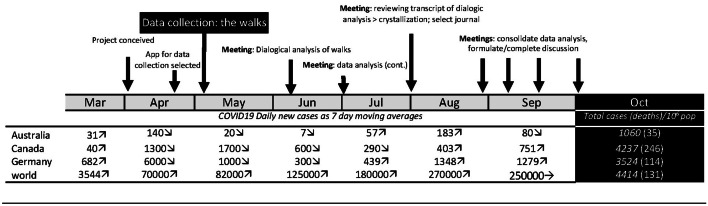
Project timeline aligned to COVID-19 data in our 3 geographic locations: Australia, Canada, and Germany with aggregated global data included for reference (Worldometer, 2020)

After we had each completed our walks, we circulated our individual maps to each other with the final instruction being to read them in preparation for collective analysis via zoom. A temporal illustration of our project indicates the time points of project inception, data collection (our individual walks), analysis and discussion, and how these align with changes in reported COVID-19 cases (new cases/day, 7-day moving average) in our respective geographic contexts (Fig. 1). When we started our collaboration, our respective countries had all made progress on “flattening the curve,” as we come to the conclusion of this particular collaboration, we are experiencing the “second wave.”

### Dialogical Processes

Wells et al. (2020) propose the term “dialogical inquiry” for the process of collective meaning-making across the disciplines, focused not on formal bracketed disciplinary exchanges, but rather the slow, emergent creation of novel knowledge through the meeting of people who embody different forms of living theory. Meetings are held for the purpose of the interpretation of texts, with specific poetics in mind: the recognition of polyphony, dissuasion of theorizing in favour of curiosity, the embodied nature of talk focused on the present rather than abstraction, and the tentativeness and avoidance of finalization (Seikkula & Olson, 2003).

#### Step 1: Basic Reflections of Maps

Meetings were held via video conferencing on Zoom. The five individual annotated maps were circulated beforehand. Each annotated map was presented by its creator, followed by 5 min of silent reflection. That person then facilitated a slow conversation, focused on the reflections of the group. This was repeated five times. These reflections were then transcribed.

#### Step 2: Crystallization

This process involved engaging in yet another collaborative dialogue, based this time on the transcriptions of our initial discussions and which kinds of combinations of images, psychogeographic text, and dialogue we might present as findings. We problematized our own constructions, highlighted vulnerabilities, reflected on positionality, and engaged in processes of mutual influence. We make no claims for their validity, instead punctuating them as what emerged at that moment in the assemblage of which we are a part.

This process was guided by the principle of crystallization. It allowed us to access multiple lived truths instead of focusing on or choosing one (Ellingson, 2009), and bring together different kinds of data, analysis, and different forms of sense making without causing contradiction (Ellingson, 2009); a “postmodern reimagination of post positivist methodological triangulation” (Ellingson, 2009, p. xii). The metaphor of crystallization was appealing, given the rich complexity of our method (collective auto-ethnography, photo-elicitation, dialogical inquiry), the many disciplines/varieties of living theory/positionalities we embody and the liminal nature of COVID-19 subjectivities in constant flux. A more woven response serves to capture our immersion, not only in the data, but in the everyday viral-semiotic-digital landscapes that we originally proposed to study. Accessing the ordinary of the fabulous and the fabulous of the ordinary mundane (Ellingson, 2009), and neglecting to situate ourselves under one method or one genre, crystallization trusts the partial, selective, and intuitive (Ellingson, 2009).

We started with the biological reality of SARS-CoV-2, not simply as a virus, but as an agent entering a complex homeostatic viral-semiotic-digital landscape, setting off a novel and ongoing reassemblage of relations of which the bodies, streets, and introspection of our walks were a part. These walks gave us access, not simply to our own potentially specious thoughts, but to narrow observations and objects or meanings that we generally take for granted. Given the absence of people on our streets, we found ourselves recording our responses to playgrounds, street signs, electronic buttons, street libraries, trees, hygiene stations, doorways, warnings, barriers, etc., in the form of urban semiotics (Gottdiener & Lagopoulos, 1986). In this human absence, material objects became the focus of our dialogue, and particularly questions regarding the multiple points of rhetoric or meaning that we might ascribe to them individually, socially, and culturally.

The crystallization follows five main objectives where condensed meaning manifests in the discussion. Around these objects, some similar patterns became evident; when confronting the experience of walking in viralscapes, we expose ourselves to affective and cognitive confrontation with the disrupted normality of everyday life and show general patterns in coping as an individual and as a group. Meeting in a group and discussing our vulnerable documentation of the experience, we reciprocally validate experiences and opinions. Overall, we document a polarization of opinion and an opinionating, meaning that objects and surroundings beforehand were mostly taken for granted, unnoticed in normality. These became emotionally and ethically loaded objects, towards those subjects relate and position. The attitudes we developed are assumptively milieu specific and come as normative loaded phenomena, which we introduced cautiously and progressively followed by continuous validation of the positions accepted by the group but metered by significant insecurity. When informally and organically agreeing on a crystallized point, each of us attached the observed in continuous comparison to former knowledge and research (e.g., attachment styles), to the self and the others in-context and in-group (we as academics, in our neighbourhood, in our nation), to former behaviour and normality, and to everyday life, or “how it used to be,” to former orders like nations and intergenerational relations. We bound the phenomenon to micro actions of everyday life, to crystallize their changing meaning(s), like walking and biking, using public transport, or using neighbourhood and infrastructure in community (like playgrounds). Characteristic for the discussion is an orientation towards an accelerated polarity and opposites. Consistently we qualify radical change, distance and closeness, trust and mistrust, former normal and new normal, politeness and impoliteness, borders and blown borders, and use emotional loaded wording of disruption, beauty, laughing, fascination, shame, greed, and stupidity. We hypothesize what has become obsolete, is no longer possible to experience due to the restrictive circumstances or changed, meaning social interaction, compliance, children, trust, hope, fun, simple unconsciousness, safety certainty, sureness, comfort, and security.

### Dialogic Introspections

**Fig. 2:**
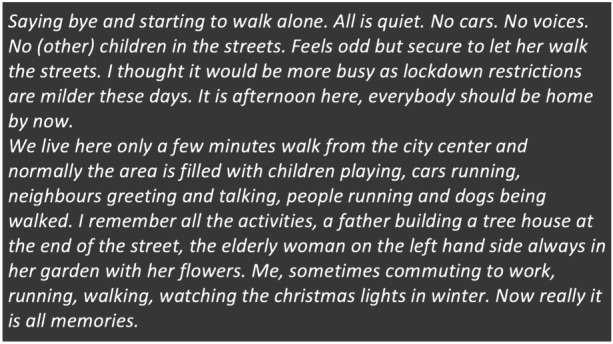
Observation and reflection from walk in Flensburg, Germany, 14 May, 2020

Setting out on our respective walks was somewhat of an adventure. Our sense of walking into a changed landscape in which we compared what we were seeing with what we had seen before is exemplified by an excerpt from Flensburg, Germany (Fig. 2).

In our collective analysis and interpretation of our respective walks, we identified five objects and themes that, to us, captured our changed relationships in the context of viralscapes, biosemiosphere, and technosphere. These objects and themes represent the crystallization of the engagement between ourselves and the environments and our collective engagement with each other in considering the maps we produced in our walks: (1) playgrounds; (2) toilet paper; (3) street libraries; (4) streets, physical location, privilege (of space) in regard to neighbourhood, and milieu but also nation; and (5) technology as eSafety. Five dialogic moments of crystallization, three objects and two broader themes, are described with an abrupt alteration and shifts in meaning.

**Fig. 3:**
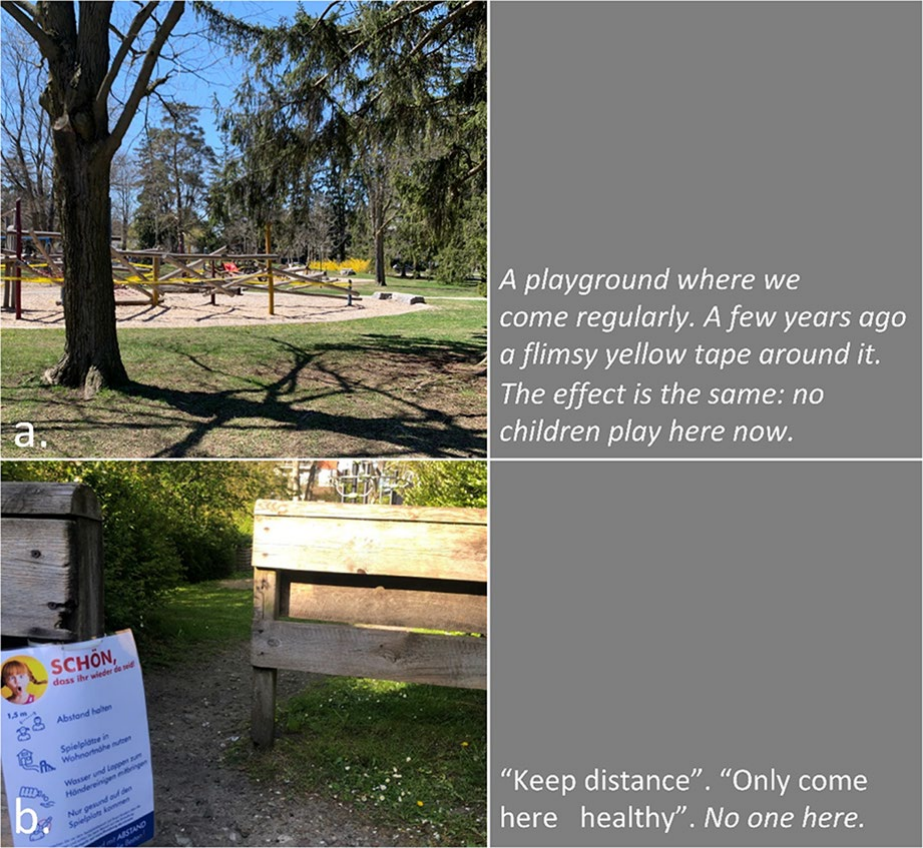
Responding to playgrounds. Excerpts from walks in Canada (**a**), and Germany (**b**) Observations recorded 13 and 14 May, 2020, respectively

**Fig. 4:**
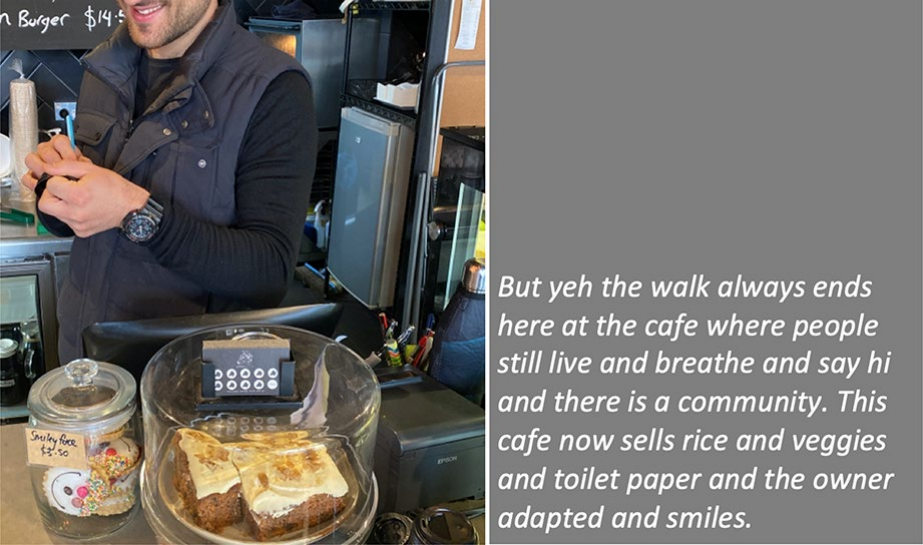
Accommodating needs: coffee, community connection, toilet paper. Excerpt from walk in Sydney, Australia. Observation recorded 13 May, 2020

**Fig. 5:**
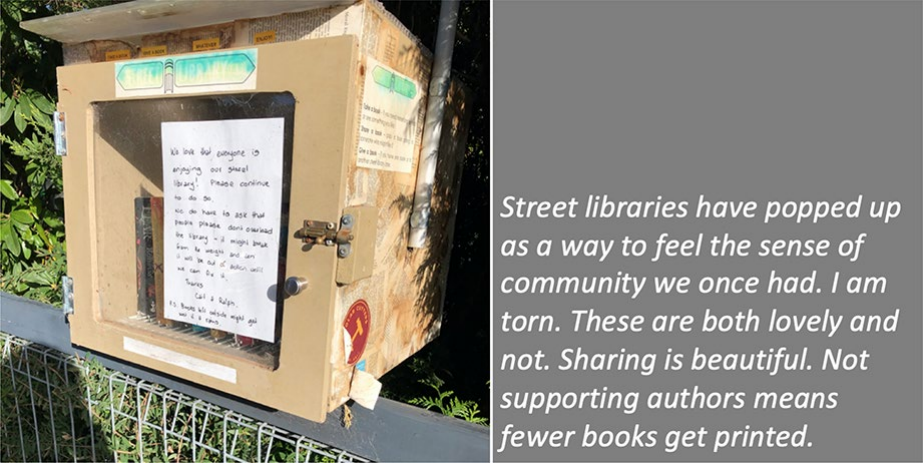
Response street library. Excerpt from walk in Australia. Observation from the Blue Mountains, NSW, Australia 16 May, 2020

**Fig. 6:**
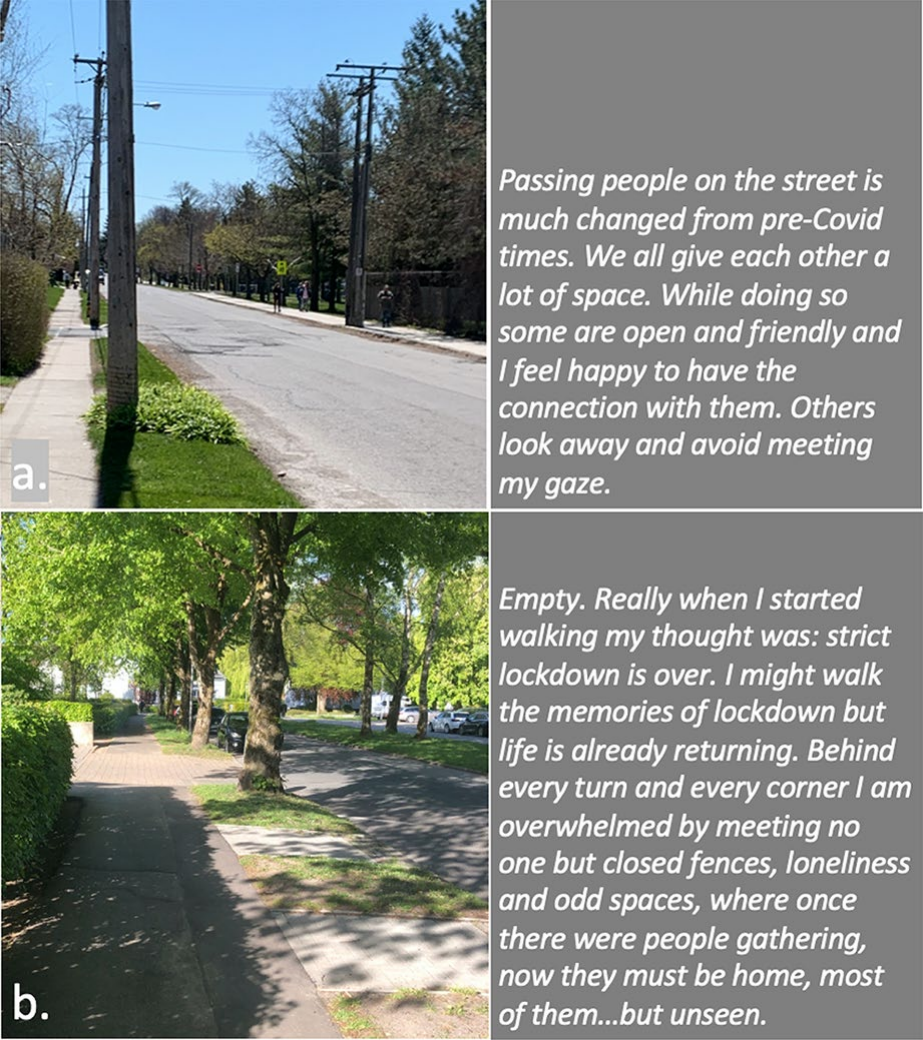
Responding to empty streets. Excerpts from walks in Canada (**a**) and Germany (**b**). Observations 13 and 14 May, 2020, respectively

**Fig. 7:**
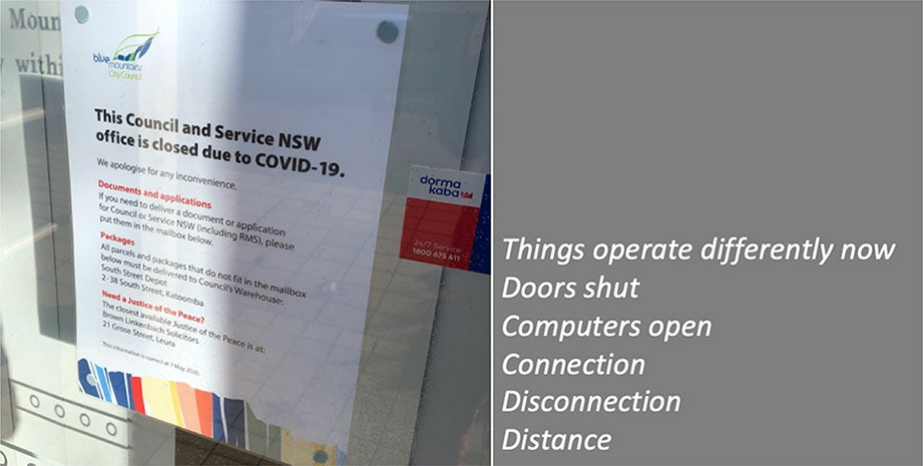
Response to locked government office. Excerpt from walk in Australia. Observation from the Blue Mountains, NSW, Australia, 16 May, 2020

**Fig. 8:**
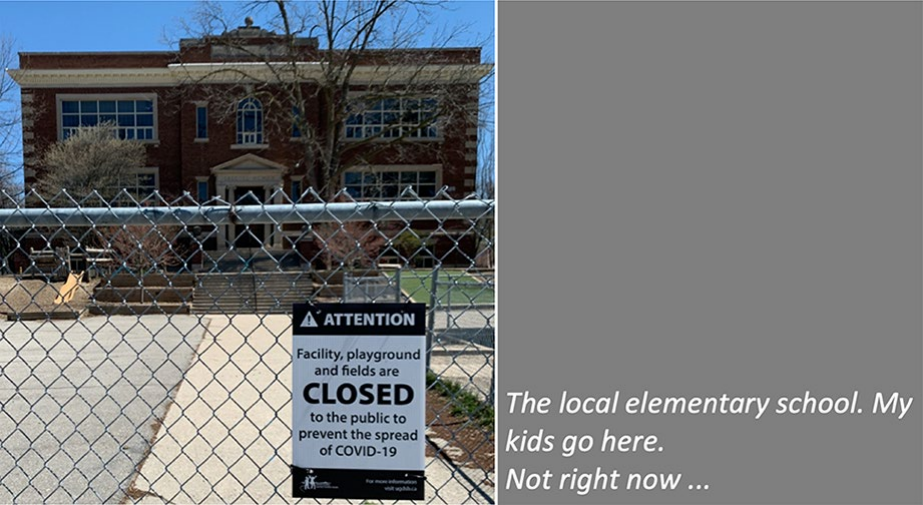
Responding to closed school. Excerpt from walk in Canada. Observation, 13 May, 2020

**Fig. 9:**
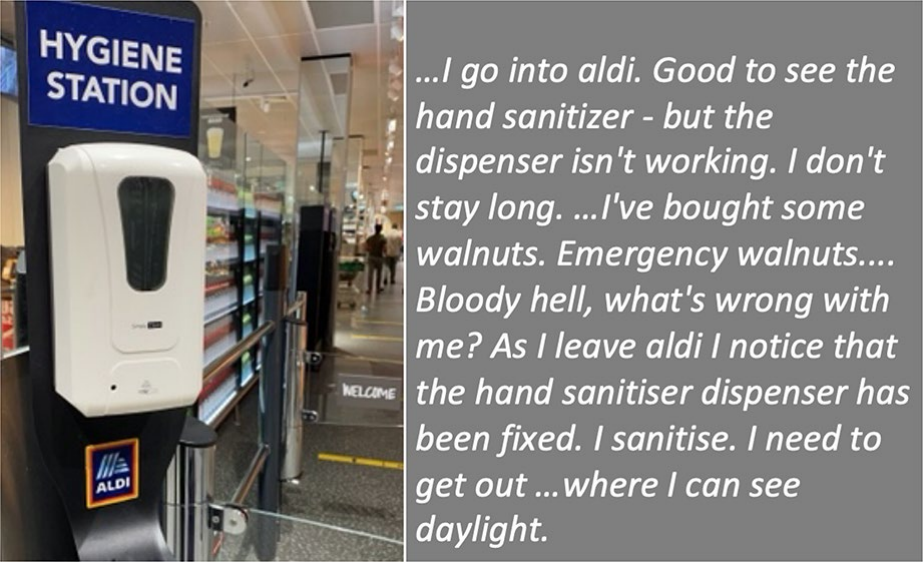
Response to ‘no touch’ hand sanitizer. Excerpt from the walk in Australia. Observation in Rockdale, Australia, 11 May, 2020

1. The Playgrounds we observed changed from being places of playfulness, livelihood, and activity to being restricted areas, soulless spaces screaming of emptiness, and silence and deadlock. Observations from the annotated walks of Kieran in Canada and Johanna in Germany catalysed our dialogue about playgrounds pictured in Fig. 3.
  *“I was thinking about the boundaries around the playgrounds. When my son was maybe around 3 years old, they were rebuilding. From one day to the next they put a fence around the whole thing. And we walked to the playground and we, and he saw the fence and he started to freak out. He started running to the fence and he saw that there was a fence but he actually ran away from us and we kept chasing after him. And he ran along this fence and you could see the distress he was in, because he couldn't find the entry. And he ran, ran and then eventually there’s no way in. I mean he was devastated, because he couldn’t get in. And now he’s seven, and my daughter is four, and so they have a very different view, developmentally they are very different. But now we saw them at the boundary tape and we explained that you can’t go in.*
  *And now my daughter is a little bit older than he was at that point in time, and she, with the other playground, pointed out ‘look the tape isn't covering everything’. So, I can play on this corner here? Really playing with this idea of boundaries. It’s more explicit for me how boundaries are negotiated all the time. And, you know, that does not mean that they are not real, but it just means that they are not self-evident. And they are partly material, partly semiotic, partly situated.”*
2. Toilet paper, in our times of COVID-19, changed from being a pragmatic, overlooked, minimum entitlement symbolizing decency, selfishness, trust, or mistrust in supply and the system and thus becoming a token of self-preservation, control, and security. Paul’s annotated walk in Australia was key for catalysing discussion about toilet paper (Fig. 4).
  *“It's animal, Livin' in the human zoo, Animal, The shit that they toss to you, Feelin' like a christian, Locked in a cage, Thrown to the lions, On the second page, If you want blood, you got it If you want blood, you got it, Blood on the street, Blood on the rocks, Blood in the gutter, Every last drop, You want blood You got it. (If You Want Blood You Got It; AC/DD, 1978.*
  *I find the toilet paper very interesting because that’s a kind of a new meme I guess in a way, symbolising selfishness, it symbolises you know responses to the apocalypse and it also comes with humour now. We have the second wave of toilet paper crisis-buying here now, it means that we are on our second wave of fear, right? It is amusing but that is perhaps how we deal with the second wave of fear. It is a second wave of panic, an affective sign, a sign of a collective feeling. And we have kind of changed what it means to panic, right? But the pictures look peaceful—and the tree is sublime, for instance. If you compare that, like in Australia we had the bushfires earlier in the year, compare the way people reacted to bushfires, it is totally different. This is a very restrained kind of fear, it’s not a usual way of expressing fear. A very private way of pulling away from everybody. We don’t really do that normally in Australia.*
3. Street libraries shift from being a symbol of friendliness and community to a potential source of risk and uncertainty, turning into a projective symbol of judging others as careless and impetuous. Gemma’s observations on her walk in Australia focused our discussion around street libraries (Fig. 5).
  *“Because lots of these little street libraries popped up…on the one hand it is an expression of community and sharing. But it's a transgression of COVID-19. You just touch all these books … anyone can walk past and touch them and then you touch them… there is no cleanliness to it … there is an assumption that the community is safe, like somehow our community is safe, but that is it. But it also hurts authors because if you share second-hand books actually you don't sell as many books so it hurts authors including local authors. I find them so interesting …there is real tension in them. I think that it does make sense with street libraries from the social perspective, I see a lot of sharing and social action too. And what I observe is that it is like the otherness of the other neighborhoods. So, like this neighborhood is not affected by covid as others maybe. So, within our safe space we can share the books and touch each other but people would say that they wouldn't leave the neighborhood because like we are more sanitized here.*
  *That ties back with the kind of refugee issue; so, I wonder if that's part of it that's being elicited in that kind of European context that we can now go back to some kind of village way of thinking.”*
4. The streets, physical location and privilege, under SARS-CoV-2 the physical location, the nation and hemisphere, the neighbourhood, and the circumstances are highly specific and determine actual danger, behaviour, and perception just as group behaviour and othering. When lonely streets beforehand were connecting simply time and space (as an empty street would hint to nighttime or Sunday), they became drawn level to symbolizing dysfunctionality and queasy feelings. The belonging to specific groups of secured income, the neighbourhoods, the collective behaviour in these neighbourhoods, the notion of world, and the options to construct a worthwhile situation in gardens, moving to even safer places and building up routines to cope make it considerably clear, that there are differences in privilege biasing the perception of the situation. Recurrence of imagery of empty streets were captured from walks (images from Canada and Germany (Fig. 6), a closed government office Australia (Fig. 7), and a closed school in Canada (Fig. 8) focused attention on what we were not seeing.
  * “If you've got a job. If you are privileged, yeah. That’s the other boundary, people who have been able to keep their employment to be able to survive. A bloke across the street lost his job, he has a partner so at least there's one income coming in, and I just thought if I lost my job what would happen? So that’s probably the really massive hole in our conversation is that we are people with jobs so we are missing the underbelly, like Johanna’s closed psychiatry unit—where are they all? Where are all the suffering people hidden away from the street. And here we are, pontificating about things. But there are jobless, domestic violence, suicides all still going but nobody knows…and those mass rallies. So, we're talking about look at these empty streets and then just in the last few weeks those streets are so not empty for lots of reasons…because you can’t keep suffering behind closed doors…*
  *I went to a cafe at Wentworth Falls…and there were people behind the counter swearing and carrying on—I don’t care if you’re a rainbow, you can’t, you still gotta, you can’t smash buildings down. Having this outrageous ‘all lives matter’ conversation. Everyone swearing. Fuck this, fuck that, the baker… the barista…And the cafe was full of elderly people. And it was this incredible breakdown and social rules—where people are allowed to be racist in public like. It was really frightening…*
  *I have narrative interviews with people who lost their companies due to covid and they are so calm. Like it took 21 years to build and I lost it in 8 weeks but really there's no anger or questioning the system or because one quick question is the lock down was necessary or the right decision if you lose everything. Somehow, maybe because the class still is a privilege nobody is questioning the decision-making and everybody is giving up on everything they have built. Their entire life. So that is something that moves me a lot. Like companies going to the ground and the COs not being too touch like, yeah, like maybe they are touched but not devastated or angry. why? I could think of many reasons to be angry. So, I am wondering a lot why everybody stays so calm…they were waiting for this …. to change what seemed unchangeable. Some kind of relief. I lost everything but at least I'm relieved that change is possible …I wonder if it is how we attribute failure. Because if my business fails because of the government making new rules of something like that, I can see people getting really angry about that. I would be so angry. But if your business fails because of volcano erupts or you know or some natural disaster, maybe it's more difficult to be angry? Maybe it’s also, ‘it wasn't my fault’ kind of relief. We're not seeing the people who lost their jobs, we're not seeing homeless people you know we're not seeing people who were in despair because I mean we know suicides are up. We know you know depression is up. We're not seeing that, right, in the landscape. And then as you were saying, also, now these streets have been filled with people protesting. And of course, we don't see the protests.*
  *Both my partners lost their jobs. I think it just depended, maybe what class you were and what country you were in. They immediately got government assistance and then just sort of saw it as a holiday. It didn't bother them very much to be honest. It was just a relief. But I think if you didn't have that consistent income, maybe like any income at all, and you couldn’t pay your rent or something and you didn't have a partner who was making lots of money like I was. Imagine if you were in Bangladesh, or… or Brazil, that’s what’s missing, isn’t it …where if you have a disability you are off the street, back to the old institutionalisation.*
  *We are studying institutionalisation and we are going in the other direction where the streets are clean of all the homeless people, disabled people, mad people. Maybe we are all, like all the rich people get to go on walks after they bake their bread… is that all the people in pain we don't want to see pain in public…particularly with domestic violence that's all hidden pain. And to expose that is the other boundary the pain must be hidden.*
  *I was talking to somebody the other day about ‘positive porn’ so in this whole notion of ‘covid’ it's only the positive stories that can come out so the ‘underbellies’, the pain, the stuff (is like you know that) that must be covered again. So covering and uncovering. I think it's striking that I get from you, especially from your pictures I get the same feeling. So I think I would have expected that this was totally different from Northern Europe and I get the same feeling. It's like you could blend the pictures; that's my feeling but maybe I'm projecting a lot into it. But I don't have that with the other Maps, for I don't know why but the streets and it's so clean and everything is so straight and so empty. I get a similar sense.”*
5. The Technology for eSafety simply arises in the context of COVID-19 with only the meaning of a niche becoming prevalent for the collective and a political two-edged sword of anxiety of surveillance, protection of the very self, and a political statement of caring for the elderly. Our collaborative dialogue focused on the introduction of covid-introduced technologies, i.e., mobile apps and hand sanitizers (hygiene stations).
  *“The Covid app is not a vaccine but the app does give me some bizarre sense of security and I’m still trying to understand why having the covid app makes me feel better because it certainly isn't going to stop me from getting exposed. When the Bluetooth Handshake happens it’s not going to protect me at all…. as we are looking at these images is the digital border. How do we relate to technology now? How porous are our bodies from machines? You know like you carry the Covid App with you like a cyborg, thinking you can become like a superhero, like an implant in your brain. Our relationship with technology has radically changed as well. …even such simple technology, like those hygiene stations where, like those ones you have the picture of in number 5 [referring to walk in Sydney suburb of Rockdale; Fig. *9*], where you don’t have to touch it to make it work you just put your hand underneath it. I think we feel most insecure when we don’t know, when we have no basis at all to form a plan of action or no basis that will, can guide us to act.*
  *It is almost better to act on a superstition than to have nothing at all. And the app, whether or not it helps, it has so many of the kind of symbols—it is backed by government, it is backed by experts, methodology…And I’m sure that there is some paranoia but there’s some very well-documented excesses and abuses both by government and private companies and I think we don’t know where we all the covid stuff is going and we don’t know what privacy rights we’re giving up in the face of covid that will not come back afterwards.”*

#### Punctuations: September, 2020

It is fascinating to look back on the writing we did 6 months ago when alongside the rising pandemic came a curiosity about new ideas that might help us to understand what was happening. The viralscape, biosemiosphere, and technosphere all served as an attempt to take snapshots of a new assemblage of relations between a myriad of changing relations instigated by the virus. This was not simply an intellectual exercise, but a means by which we hoped we might both understand and resist the fear we experienced, in the liminality of a new and unknown reality. This fear included the awareness that as humans we were not as central as we might have thought, but that an invisible virus might remind us of our place within a myriad of non-human agents, including those in microbial, animal, cultural, and digital realms.

This project aimed to see how we might resist dehumanization while accepting the new ontological relations that were becoming. We mobilized method as the means for this resistance: firstly by mapping our internal landscapes while walking through external ones, changed through lockdown and desolation; secondly by engaging in slow dialogue, in a social environment gone digital. Would this curated, operationalized walking and talking provide a means by which we might reassert our humanness and momentarily find our place in the assemblage?

Each individual presents a punctuation on thoughts and conversations that continue. As a marker of humanism, we return to our thoughts as individuals.

#### Paul: 11 September 2020 9.35 am

“I gave a talk the other day on this piece of research, and others have become engaged in since the pandemic, in front of a large group of empirical scientists. The Covid Colloquium. Some of them presented on interesting topics, such as gambling under lockdown, how sex has changed, issues relating to racism. I found this project to be much more personal; something I needed to do to make sense of my own life and emotions, through the pursuit of new ideas and a retreat into the joy of trying new research methods.

It seems clear to me that we were able to both capture and theorise on our relation with non-human objects, given our findings crystalised around a specific set of objects, given cultural meaning by us, and therefore ascribed agency through semiotic means. I assume if the pandemic continues and we maintain our distance from others, these processes, that have always been there, will be cast into further relief. Because of the virus and through our methods our affective-hermeneutic relationships with the built environment, road signs, barriers, libraries, toilet paper and apps could be explored.

From my own perspective this process did ease my distress, given it provided a means by which I could draw on new theoretical resources I had not previously known, as we read and had dialogue about actor-network theory, semiotics, prometheanism, affect theory, the microbiome and other concepts, all taking their place alongside objects in the shifting assemblage. More than that, however, was the realisation that belonging, affection, respect, and the joy of relationships could all be possible in a two-dimensional environment. I fondly remember Rosanne starting our 6am meetings in the dark so we could see the sunrise through her window, or Gemma showing us her arm after surgery. I recall the thrill as Kieran introduced us to the concept of crystallisation, and the fond memories seeing Johanna run in Vienna in the middle of winter.”

#### Gemma: 1 October 2020 9:15 am

“As Australia, and the world, moves slowly towards liberation from the grip of COVID-19, I’m still struck by what Mol (2006) calls the ‘multiplicity of norms’. There is no normal anymore, instead we are a messy, clashing mix of normalities and abnormalities, old and new normals. Living through a time of flux means we cling to whatever stability we do have in order to remain grounded. In many ways this group has provided me with a sort of stability, a regular 6am touch point and check-in. It gave me a chance to step back from the comparative chaos of redesigning courses for the online environment, and reflect on the realities of place, space and being with highly attuned and creative colleagues, only one of which I’ve met in the flesh.

I was unfortunate enough to have a family member fall ill from COVID-19 during the aged care crisis in the state of Victoria, Australia. Thankfully they pulled through, but while it was still touch and go this project provided a way to think about the crisis—to widen my scope of analysis and observation thanks to Paul and Rosanne, then crystalize it guided by theoretical frameworks from Johanna and Kieran. There are very few projects that would leave me excited to wake up at 5am for meetings in the dark for. This paper is a testament to the intellectual nourishment that collaborative interdisciplinarity can bring, and a reflection on the quality of Kieran, Johanna, Rosanne and Paul as researchers, and as people.”

#### Rosanne: 5 October 2020 11.41 am

“Teaching during Australia’s first wave was intense. I had had the plants I use in teaching delivered to my front garden so that I had them on hand for classes. At the end of the semester I was invited to present how I successfully managed to teach online at a number of fora. The expectation of offering only positive narratives is itself exhausting. For me it was critical to get real about how hard this had been. At the end of many of my three hours online practical classes I had sobbed with exhaustion. I sobbed before being interviewed by someone in the Faculty wanting a good news story on teaching during Covid lockdown. Working on this paper provided much needed intellectual and creative stimulation. I am grateful that the walk I took brought my dad back into focus; he would have been 100 today. It has been a delight being able to share the difficult parts of the job with the co-authors, and I find it so interesting that I have only physically met Paul. A pivotal moment for me while we were writing this paper was being introduced to the work of Bakhtin and his concept of “many speechedness”, this certainly is enacted in the narratives from our individual walks, our discussions and the how we have crafted this paper. “Many speechedness” resonantes with how we present ourselves in our academic work—different facets of ourselves revealed by shifts in what we say, and what we don’t say.”

#### Kieran 9 October 2020 11:11 am

“When COVID-19 hit, I was not intending to do research on the topic. Clearly, I saw the need for research from a social sciences and humanities perspective to balance the expected prioritization of biomedical research on the agents of this crisis. But from the beginning I felt COVID-19 would not make all the other problems and challenges in our lives go away. It might exacerbate them, temporarily divert attention from them, but surely it would not do away with those other problems. And, therefore, I felt justified in resisting my research attention being drawn, along with my attention in personal and professional (teaching) life, into the maelstrom of COVID-19. In the words of intergalactic colonizers, my resistance proved futile, and before long I was heavily engaged in research on people’s responses to COVID-19 and associated risk communication, people’s perspectives on an eventual COVID-19 vaccine, and developing deliberative processes to create mechanisms for bringing lay people into dialogue with policy makers in considering values and implications of societal responses to COVID-19. When Johanna invited me to join this group to work on psychogeographies of COVID-19 I was completely overwhelmed and felt that one more project might break me. But the opposite turned out to be the case. Our regular video calls as a research and writing group were inspiring and personally fulfilling. It quickly became apparent that with each other we did not need to present a positive story about our engagement with COVID-19. Frustrations we experienced in our personal and professional lives around particular stages and challenges of COVID-19 life could be expressed authentically and met with understanding and humour, a foundation of dialogue on which to build our scholarly work together. Looking back on the time during which we created our maps is strangely romantic in its own way. At the time the world felt alien and strange; but now the world feels far more alien and threatening to me, such that those early days of COVID-19 have taken on a feeling of familiarity and even comfort. As preoccupation with the virus has changed, attention in many contexts has intensified on the political ramifications of particular governmental and institutional policies with regard to COVID-19. I have not heard the previously common phrase of “we’re all in this together” for some time, and I believe that now (at least in my social and cultural surroundings) it would ring hollow and somehow forced and inauthentic. All of this emphasises for me the transience of knowledge about human phenomena. Our engagement with COVID-19 landscapes over the last few months has produced different meanings at different times. Our insights, in this regard, are historical, just as Gergen (1973) argued all of psychology is, but here perhaps so much more evidently.”

#### Johanna 10 October 2020 1:16 pm

“Identifying strongly as scientist and as psychologist the reflections below actually never crossed my mind, and that was a shortcoming, a fragmentation misplaced and doing harm to my ability of understanding subjects and their psyche.

When wandering the jungle of Thailand I was questioning my relationship to nature within the Humboldt paper (Degen et al., 2020). I wondered about humanity, who are we and how severely damaging and unromantic in all facets are the effects of our being here? Dominantly I was mistrustful from a point of morality: nature is better off without humans. The planet’s condition made me question human humanity towards all other living things. Only a split second of a few months later a shift, not only in the hemisphere, but regarding my notion of the world was forced by Covid-19. Due to the power of embodied knowledge and emotions my cognitive reflections were urged to make a severe change in perspective. I today not only mistrust the humankind, today I additionally mistrust nature as a possibly dangerous, threat filled environment, not only the romanticized nurturing source of peace, curing depression, an objective we have to protect from us, but like turning a switch changing the power. Just like that nature is revealing a threatening face itself, causing eco anxiety and uncertainty about a future in general, the end of romance. Are we doomed? Is this Kali Yuga? The era of chaos and devastation? When I was wandering the jungle, I felt nature was threatened by humanity, today humanity is threatened by nature. What a relief. I am anxiously cheering for nature and at the same time, as a psychologist, I help the subjects turning to me for help. They come not only with personal challenges like eco anxiety, but mostly with broken relationships in times of distress and pressure: do we like each other even though we spend time together?”.

## Covid-19: Technological Amputation and Mental Cohesion (Date: 7 May, 2021)

In the disruption of everyday life under the rise of the pandemic, we were zoomed into the technosphere and our bodies became isolated and amputated. Our minds were predominated by distress under restrictive conditions, perceived deficiencies, and a constantly and abruptly changing order and meaning. We accessed the study of socio-spatial conditions from a first person’s micro perspective, revealing that the global change, made a change within the very individual revealing meaning that probably is valid on the macrolevel as well. If so, it means that the circumstances during the pandemic generally lead to disruption of everyday life and meaning, vulnerability of the individual, opinionating and othering through comparison and judgement leading to polarity and opposites, emotional loaded wording, and changed meanings of usually stale symbols.

Taking the micro perspective, as first-person perspective and the individual context, as a starting point, we developed a collective and tech-enabled long distance, healing process of coping, where cohesion, rhythmic movement, stabilizing routines, and productivity guided us towards an integration of positive possibilities in the otherwise dystopian scenery.

As a group, we reinterpreted the disadvantage of being bodily apart to a positive equalization of relationships, where it becomes redundant where and when subjects are on a global level, where distance and time become meaningless players in a digital place.

Getting together was less about another Zoom meeting, it was overcoming the characteristics of the online meeting. It developed power through counteracting the reduction to the cognitive and the one-dimensional channel of serial communication. First though the entanglement of physical location, bodily movement, shared personal artefacts (in the form of pictures), and intersubjective commitment to each other, our mental dialogue transitioned from being exhausting to being energizing.

Moving through the Viralscape became a group activity, connected through imagination of the others walking, we focused to contributing to the collaborative hub of material. Through this hub we were able to drag real-life experience and geographical location into the digital. There the material became the vehicle to dip into our colleagues’ multiple dimensional experienced and re-living situativity together. In that dialogue, we were able to recognize similarities and confront presumptions and projections to carve out the inherent meaning of our observations.

In that space, it was possible to experience the self of being capable, belonging, and productive. That way the project turned into a sort of group coping and healing. In rapid change, it became a guiding help to establish a reliable structure in a collaborative group connecting against the feeling of being sold out. Together, we stayed with trouble by confronting the context of the virus and move through it with eyes wide open. Through a reflexive reinterpretation of options, we could enrich the exhausting cyborg technological experiences into an empowering, healing, loving and visionary mode of belonging, engagement, and productiveness as a form of stabilizing and empowering coping.

## Footnotes

1 “Similarly, according to Kafka (1950) and De Rivera (1977), there exist four basic emotional movements: moving oneself “toward the other” (e.g., affection, mourning), moving the other “toward oneself” (e.g., desire, greed), moving the other “away from oneself” (e.g., disgust, anger), and moving oneself “away from the other” (e.g., fear, disgust). The four are related to the gestures of giving, getting, removing, and escaping. These basic movements are connected to a bodily felt sense of expansion or contraction, relaxation or tension, openness or constriction, etc.” (Fuchs & Koch, 2014, p.4).
